# 3D-Printed Chips: Compatibility of Additive Manufacturing Photopolymeric Substrata with Biological Applications

**DOI:** 10.3390/mi9020091

**Published:** 2018-02-23

**Authors:** Megan Carve, Donald Wlodkowic

**Affiliations:** 1 School of Science, RMIT University, Melbourne, VIC 3083, Australia; s3437621@student.rmit.edu.au; 2 Centre for Additive Manufacturing, School of Engineering, RMIT University, Melbourne, VIC 3083, Australia

**Keywords:** lab-on-a-chip, bioassay, toxicity, additive manufacturing, polymers, 3D printing

## Abstract

Additive manufacturing (AM) is ideal for building adaptable, structurally complex, three-dimensional, monolithic lab-on-chip (LOC) devices from only a computer design file. Consequently, it has potential to advance micro- to milllifluidic LOC design, prototyping, and production and further its application in areas of biomedical and biological research. However, its application in these areas has been hampered due to material biocompatibility concerns. In this review, we summarise commonly used AM techniques: vat polymerisation and material jetting. We discuss factors influencing material biocompatibility as well as methods to mitigate material toxicity and thus promote its application in these research fields.

## 1. Introduction

Additive manufacturing (AM), colloquially known as three-dimensional (3D) printing, is an automated computer-assisted design (CAD) and fabrication method developed in the 1980’s [1]. Via AM, structurally complex monolithic devices can be built from a range of materials, such as liquid-polymer or powder grains, with various material, mechanical, and physical properties. AM is a versatile and agile technology platform suited to the rapid development of prototypes and the fabrication of adaptable high-value products. Expansion of innovative desktop AM systems, exhibiting fast build-speeds and improved printing resolutions, coupled with decreasing prices of AM technologies, is enabling their rapid adoption in small-scale industries and laboratories. By 2020, the global AM industry revenue is predicted to exceed $21 billion [2]. It’s growing popularity is due to the advantages it has over traditional ’subtractive’ or ’formative’ manufacturing techniques, which rely on economies of scale and are comparatively wasteful [3].

Demand for inexpensive prototyping and fabrication of lab-on-a-chip (LOC) devices has stimulated substantial interest in alternative fabrication methods. In this regard, AM has attracted interest within the LOC community, which aims to reduce the cost and complexity of prototyping and developing bespoken devices [4]. AM circumvents cumbersome processes inherent to traditional manufacturing methods (e.g., photolithography), such as complex multi-step fabrication techniques and the use of expensive clean room facilities. Specifically, the vat polymerisation AM methods, stereolithography (SLA), multi-photon polymerisation, and digital light processing (DLP), provide elegant fabrication alternatives.

**Figure 1:**
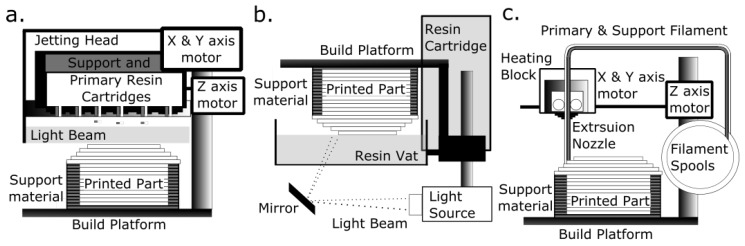
Examples of additive manufacturing (AM) systems that use polymer materials: (**a**) Material jetting, where a photo-sensitive photopolymer is deposited in droplets that are then polymerized by a passing light source. The process is repeated so that the solid object is built up layer by layer on the build tray. (**b**) Vat polymerization, where a photosensitive polymer contained in a vat is polymerized in a layer-by-layer fashion either by a light-beam (stereolithography and multi-photon polymerization) or by light-projection (digital light processing ). (**c**) Material extrusion, where the polymer filament is softened as it passes through a heating block and extruded in a layer-by-layer fashion.

Vat polymerization AM can produce optically transparent structurally complex monolithic devices with functional elements such as integrated fluidic interconnects, valves, pumps, and lenses [5]. In this method, a solid object is built in a layer-by-layer fashion by the selective exposure of a photosensitive polymer resin to a focused laser beam or light projection (Figure 1) [6]. Presently, most consumer-grade 3D printers build objects by a process known as fused deposition modelling (FDM). This involves melting and depositing a filament of plastic material such as acrylonitrile butadiene styrene (ABS) and poly(lactic acid) acid (PLA) in a layer-by-layer fashion. Decreasing costs of vat polymerisation machines means they are no longer only affordable to large-scale industries but are accessible to the individual consumer.

A critical limitation of AM systems for LOC applications, however, is the printing resolution. In general, AM systems are capable of producing a minimum printable feature size 70–300 μm and a *z*-axis layer height of >20 μm [1,7]. While current generation AM systems appear less suited to the fabrication of true microfluidic systems, they are suited to the fabrication of milllifluidic or mesofluidic devices, the latter is characterised by a channel’s cross-dimensions ranging from 250 μm to 2000 μm and high aspect ratios. Design and structural constraints make them particularly difficult to fabricate using conventional photolithography techniques, thus demanding non-standard fabrication approaches ideally suited to AM [8,9,10]. Emerging applications in biomedical research and ecological toxicity testing include in-situ analysis of embryos and small metazoan organisms [11,12,13,14,15].

Despite the perceived advantages, reports on the toxicity of AM photosensitive polymeric materials have curtailed their application in AM-LOC technologies aimed at in vitro bioassays and biotests [13,16,17,18,19]. Resin compounds such as photoinitiators (PIs), monomers, short-chain polymers, and auxiliary compounds may leach from final printed parts in aqueous media and consequently may compromise part biocompatibility. Furthermore, attempting to eliminate these potentially toxic compounds by following standard manufacturer-recommended part-cleaning procedures might not adequately mitigate toxicity. This review focuses on the perceived toxicity of AM photosensitive polymeric materials as well as current approaches to alleviate toxicity, with the aim of facilitating applications in biomedical and biological research.

## 2. Overview of Additive Manufacturing (AM) Fabrication Technologies

AM technologies are classified according to the initial condition of the build material and the physical principles underlying the solidification process (Figure 1) [1]. In 2017, photo-polymerisation AM, which encompasses material jetting (MJ) and vat polymerisation (SLA and DLP), accounted for the majority of materials used in the global AM market [20].

The vat polymerization process produces a solidified 3D object by localised irradiation and polymerisation of a light sensitive resin (contained in a vat) by a spatially controlled light source [1]. The light source used in SLA is a spot laser that irradiates the resin as it scans in an x–y direction of each plane, whereas the light source used in DLP is a projector and the whole x–y plane is irradiated in a single flash. For both, the final object is built in a layer-by-layer fashion as the build platform moves through the resin along the *z*-axis, whereas multi-photon, also known as two-photon, polymerisation (MPP), produces a solid object by focusing a femtosecond laser beam into the resin vat. The laser moves in any direction within the resin, so it is not a layer-by-layer technique [21,22].

In contrast, MJ is a process similar to inkjet printing and involves jetting droplets of photosensitive polymer resin onto the build platform (Figure 1). Resin is solidified by a passing light source, and the process is repeated in a layer-by-layer fashion, as the object is built from the bottom up. Vat polymerization technology, more than MJ, can accelerate micro/milllifluidic device development since it is capable of achieving finer resolutions. It has been used to build LOC and micro-devices from optically transparent materials [5,23,24,25].

SLA and DLP has been used in LOC design and manufacture. The authors of Au et al. [26] built a peristaltic pump and cell perfusion chamber with integrated fluidic valves, and the authors of Comina et al. [27] built a monolithic LOC with integrated check-valves. In addition, LOC devices have been fabricated via replica moulding in poly(dimethylsiloxane) PDMS from moulds [28,29]. SLA and DLP are applied in dental implant design and production, surgical planning, the building of anatomical prosthetic devices, and tissue engineering [5,30,31].

MPP is suited to the design of microfluidic devices, as it can build structures down to 200 nm from materials classified as biocompatible [32]. It has been used to build micro-needle arrays for vaccine and drug delivery [33] and develop complex suspended micro-channel resonators for LOC biomechanical sensing applications [34] as well as integrative optofluidic refractometers with microtubes of various diameters and wall thicknesses [35].

## 3. Photopolymerization and Stereolithographic Resins

Polymerization, by chemical reactions, connects unsaturated monomer molecules to propagate a polymer-chain network [36,37]. To create a 3D solid object, a photo-sensitive stereolithographic polymer resin is solidified by the successive vitrification of the photopolymer by a light-initiated chemical reaction that leads to the cross-linking of polymers by produced reactive species (free radicals, cations or anions) [38]. The photo-polymerisation process is consistent with ‘chain-growth’ polymerization in that it requires initiation, propagation, chain transfer, and termination [36,37,39].

Resin components include a photo-sensitive polymer and a photoinitiator system along with other additives and fillers, such as inhibitors, plasticizers, light stabilizers, and pigments (e.g., shown in Table 1, Table 2 and Table 3). Auxiliary compounds may improve polymerisation (in the industry termed ‘curing’) efficiency and enhance mechanical, physical, and aesthetic properties of the final printed part. Ultimately, final material properties depend on the post-cleaning and post-curing process.

**Figure 2:**
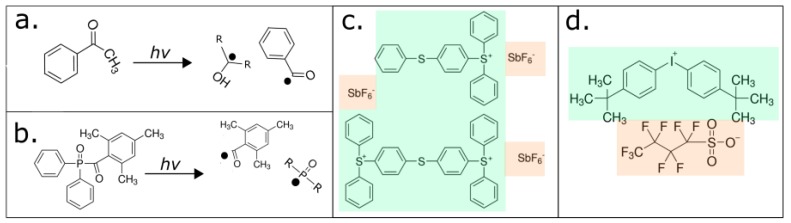
The two photoinitiator (PI) mechanisms are radical (**a**,**b**) and cationic (**c**,**d**). In the radical system, absorption of light (*hv*) produces a free radical by homolytic cleavage, propagating polymerization, for example, as in (a) hydroxyacetophenone and (b) phosphine-oxide, where R represents a methyl group in (a) and a phenyl ring in (b). In the cationic PI system, in PIs such as (c) triarylsulfonium hexafluoroantimonate salts and (d) bis(4-tert-butylphenyl)iodonium perfluoro-1-butanesulfonate, light is absorbed causing heterolytic and homolytic cleavage that forms a cationic portion (labeled in green) and anionic portion (labeled in pink). The generated molecules are reactive with monomers, forming an acid and free radicals, propagating polymerization (open source image adapted from [36]).

In radical polymerization, photolytic energy is absorbed by the PI and the produced reactive species is a free radical, a molecular fragment having one unpaired electrons (Figure 2) [36]. The propagating site of reactivity is a carbon radical [40]. Free radicals are transferred from monomer to monomer during chain growth. Radical polymerization terminates when two radicals, either the forming polymer chains or PI fragments, combine.

In cationic polymerization, photolytic energy is absorbed by the PI to release free radicals and a Lewis or a Brönsted acid [40,41]. The propagating site of reactivity is a carbocation. Charge is transferred from monomer to monomer during chain growth. Termination occurs via unimolecular rearrangement within the counterion, i.e., where an anionic fragment of the counterion combines with the propagating chain end, inactivating the growing chain, and reducing the concentration of the initiator complex [42].

Two polymerisation steps are required to produce the final object built using vat polymerisation AM [31]. The first-step occurs in the vat of the machine and is by the light emitted by the machine. This step produces a partially cured object, referred to as ‘green’. While the object at this stage has its final shape and form, a second curing step is need to optimise its mechanical properties. Typically, the ‘green’ object is first rinsed in a solvent to remove polymerised resin. The second curing step occurs in a purpose-built ‘post-curing’ oven that emits a (stroboscopic) light at a wavelength that should be matched to the resin type. The object typically must be systematically re-orientated during this step, so that exposure of each surface of the geometry to the light source is maximised.

### 3.1. Photoinitiator Systems

A PI compound (examples are shown in Table 1) acts as catalyst for photopolymerisation by converting absorbed light energy into chemical energy, free radicals, and/or cations [43]. To enhance the efficiency and depth of polymerization, stereolithographic resins will typically contain multiple ‘PIs’ and a thermal initiator compound. A thermal initiator, such as benzoyl peroxide or Lauroyl peroxide, ideally does not affect the resin during printing, but operates during ‘post-curing’ to aid in interior part polymerization. Radical PI systems are classified by their mode of operation as either a Norrish Type I or Norrish Type II. The Norrish Type I PI molecule undergoes homolytic cleavage to produce free radicals upon the absorption of light [38], whereas the Norrish Type II PI is a bimolecular system, where absorption of light, typically ultra-violet (UV)-C, causes a light absorbing molecule (or sensitizer) to interact with a second molecule (a co-initiator) to generate free radicals [44].

A cationic PI system consists of a cationic and anionic pair, and each component has an explicit role in the polymerization mechanism [37]. The cationic portion of the PI molecule absorbs light to produce an excited electron state, whereas the anionic portion of the PI molecule absorbs light to produce an acid [41]. The excited electron state causes the PI molecule to undergo homolytic cleavage to yield several free radicals and heterolytic cleavage to produce another cationic species and an acid. Typically, cationic PIs are either sulfonium or iodonium salts (e.g., 4-octyloxy-phenyl-phenyl iodonium hexafluoroantimonate). Cationic photopolymerization is used for monomers, such as epoxides and vinyl ethers, which cannot be polymerized by free radical mechanisms [45].

For photopolymerization to proceed efficiently, the absorption wavelength of the PI must overlap with the emission spectrum of the light source and there must be minimal competing absorption by the other resin components [46]. Optimal absorption of each PI occurs at a specific wavelength range, for example, phosphine-oxide compounds will typically have an absorption maximum between 360 nm and 400 nm, and camphorquinone has an absorption maximum at a wavelength of 468 nm [30]. Catatonic PIs absorb in the wavelength range between 220 nm and 280 nm, with the different molecular configurations around the sulphur or iodine atom determining the specific absorption maximum for each PI [39].

### 3.2. Photopolymer Matrix/Systems

Photopolymers are mono-, di- and trifunctional monomers, acting as cross linkers, and hyper-branched oligomers that are multifunctional, acting as both cross linkers and diluents [57]. Classical photopolymer systems contain a single type of photopolymer (e.g., acrylic, epoxide, vinyl-ether, or a thiol-ene), whereas commercially available resins have combinations of photopolymers to enhance mechanical properties of the final fabricated object and to overcome the limitations of single polymer systems (e.g., low strength and high anisotropy). Examples of photopolymers are shown in Table 2.

Most free-radical curing resins employ the unsaturated functionality of acrylate and methacrylates; however, allyl- and vinyl-based formulations are also available [1]. An acrylate oligomer backbone can be modified to be of a different polymer class to achieve the material properties desired in the final object. For example, material properties are customized when an acrylate oligomer is combined with highly flexible polybutadienes, rigid and chemically resistant bisphenol A epoxies, and flexible yet tough polyurethanes [58]. Polyester- and acrylic-based acrylate oligomers are also commercially available.

The biocompatibility of candidate photopolymeric materials for the AM of biomedical devices has been carefully scrutinised. Organically modified ceramic (ORMOCER) composite resins, for example, have been used with MPP and SLA to build devices including scaffolds for tissue engineering, microneedles for drug delivery, and dental implants [33,59,60]. ORMOCER-based photopolymer resins contain silicon alkoxides, organically modified silicon alkoxides, several metal alkoxides and in/organic monomers [61]. Typically, they operate either as methacrylate alkoxysilane systems, initiated via radical polymerization, or epoxysilane systems initiated via cationic polymerization. The biocompatibility of ORMOCER copolymer resins increases as the content of inorganic co-polymers decreases, such that leaching of toxic residues is minimised [62]. For example, a polymerized object made from an ORMOCER-based resin may contain up to 50% unreacted methacrylate monomer. These are liable to leach in an aqueous medium and cause toxicity.

Polyethylene glycols (PEGs) are polymers of ethylene oxide [63]. PEGs are non-toxic, except when they are administered at exceedingly high doses (for humans, >10 mg/(kg·day^−1^)). Material properties of PEGs can be fine-tuned by modifying the molecular weight (MW) of the PEG backbone and by cross-linking them with acrylate groups. Polyethylene glycol diacrylate (PEG-DA) contains double-bond acrylate groups at each end of the PEG chain.

PEG-DA is a relatively biocompatible material [64,65]. While PEG-DA is used in biomedical applications, toxicity of PEG-DA can arise from unreacted monomers and PIs. Consequently, assessment on biocompatibility precedes each application [64]. The authors of Urrios et al. [66], for example, built transparent microfluidic devices in PEG-DA (MW 250) using SLA. To mitigate the toxicity of unreacted PEG-DA monomers and PI, the surfaces were subjected to an additional curing step in a UV bath to allow residual compounds to leach. The authors of Traore and Behkam [67] combined PEG-DA hydrogels with photolithography to produce LOC devices for use in cellular assays. To mitigate toxicity, gels were soaked in PBS overnight to remove excess PI and unreacted monomers.

Direct AM of PDMS is still in its early stages of development. The authors of Femmer et al. [68] used AM to create the first membrane made from PDMS using DLP. They fabricated features in the range of 150 and 300 μm from a custom PDMS-based resin. However, the optical clarity of the material was reduced because PI concentration and biocompatibility assays were not undertaken. PDMS materials have been applied, to a degree, in the development of microfluidic LOC using AM. The authors of He et al. [69] produced an LOC using FDM, sugar, and PDMS. Micro-channels were created by extruding melted sugar on a PDMS-based layer. PDMS was cast over the sugar layer and the sugar was removed with water. The authors of Comina et al. [29] used AM master moulds to cast PDMS into LOC. In addition, PDMS has been used to coat AM LOC to improve biocompatibility.

Additives and fillers are auxiliary compounds included for the purpose of enhancing and customizing qualities of the printed part. For example, vertical print resolution and transparency is improved, and solubility is reduced, by inclusion of ‘light-blocker’ compounds, such as carbon black and Naphthol-based pigments, and UV blockers, such as 2,2’-(2,5-thiophene diyl) bis(5-tert-butyl benzoxazol) Liu and He [82]. All acrylate-based resins require auxiliary compounds to extend resin shelf life. Inhibitors, which are usually a quinone-based compound such as butylated hydroxytoluene, hydroquinone, hydroquinone monomethyl ether, and benzoquinone, prevent spontaneous polymerization during storage [83].

In general, commercially available resins utilize a wide range of auxiliary compounds that serve various purposes such as tuning material, mechanical, physical, and aesthetic propertis. Several examples of auxillary compounds are shown in Table 3. Further example compounds include silica, titanium dioxide, zinc, iron oxides, silver, silicon nitrides, calcium, lead, cerium, tin, zirconium, strontium, barium, borosilicate glasses, kaolin, quartz, talc, rubber impact modifiers, thermoplastic and cross-linked polyurethanes, polyethylene, polypropylene, polycarbonates, and poly-epoxides [83,84,85,86,87,88,89].

## 4. Compatibility of AM Substrata with Biological Applications

Biocompatibility of polymeric substrata used to fabricate LOC technologies is critical for any biological and biomedical applications. Bioassays on living cells and tissues are conducted in aqueous media and leaching of any chemicals from 3D printed plastics after contact with water can directly affect test specimens. Until recently, critical biocompatibility issues and potential hazard risk implications of widespread usage of 3D printed polymers have received only marginal attention [13,17,18,19,56,90]. Data on toxicity, bioaccumulative potential, persistence, and degradability of photopolymer materials is largely lacking [4]. This is in part due to a lack of available information on resin composition, which constrains independent evaluation of environmental and human health risk.

Typically, commercial resin formulas are proprietary. Only known hazardous materials are declared in material product safety data sheets (SDS). Compounds considered by the manufacturer as posing no significant risk are omitted. Open source and patent documents showed limited insight on the potential formulas and compounds used in resins. This indicates that formulas are complex, potentially containing greater than 20 compounds [82,83,84,85,86,87,88]. Consequently, it is difficult to predict which compounds may leach and to estimate and measure the toxicity of individual and mixtures of compounds. Comprehensive biological and chemical testing is a consequence necessary to evaluate biocompatibility.

Generally, substrata used for MJ and vat photopolymerization AM can have greater toxicity than those used in material extrusion AM [17,91]. Typically, photopolymerization AM is reliant on acrylate- and methacrylate-based compounds, which have up to a 90% weight-to-weight ratio (*w/w*), and phosphine-oxide-based PI systems, which are less than 5% *w/w* (Table 1 and Table 2). These compounds are known to be acutely and chronically toxic to aquatic test organisms, including fish, algae, and water microorganisms. In mammals, chronic exposure to these compounds has sublethal effects that include impaired reproductive, liver, and kidney function and abnormal growth and development. Despite this, studies investigating AM polymer toxicity have primarily focused on the emission hazards of fumes resulting from material extrusion techniques, and photopolymerization materials have been largely ignored [12,13,18,92,93].

The two principal mechanisms by which compounds can be released from a polymerized object are (1) degradation or erosion and (2) extraction by solvent or aqueous medium, the latter often referred to as a leaching of toxic residues [91]. Erosion, which may be caused by photo, thermal, mechanical, or chemical factors, and the solubility of the resin-matrix also influence the release of compounds. For example, UDMA-based resins (urethane dimethacrylate, 1,6-bis(methacryloyloxy-2-methoxycarbonyl amino)-trimethylhexane) are less water-soluble than materials containing bisphenol-A-glycidyl methacrylates and are thus more susceptible to erosion [91]. Salivary esterases can erode the surfaces of resin-based dental materials, releasing methacrylic substances that subsequently enter the intestine via swallowing, diffuse into the circulatory system, and remain in the body until metabolized [94]. Unpolymerized co-monomers, triethylene glycol dimethacrylate (TEGDMA) and 2-hydroxyethyl methacrylate (HEMA), released from polymerized parts used as dental implants, have been detected in the human circulatory system [95].

**Figure 3:**
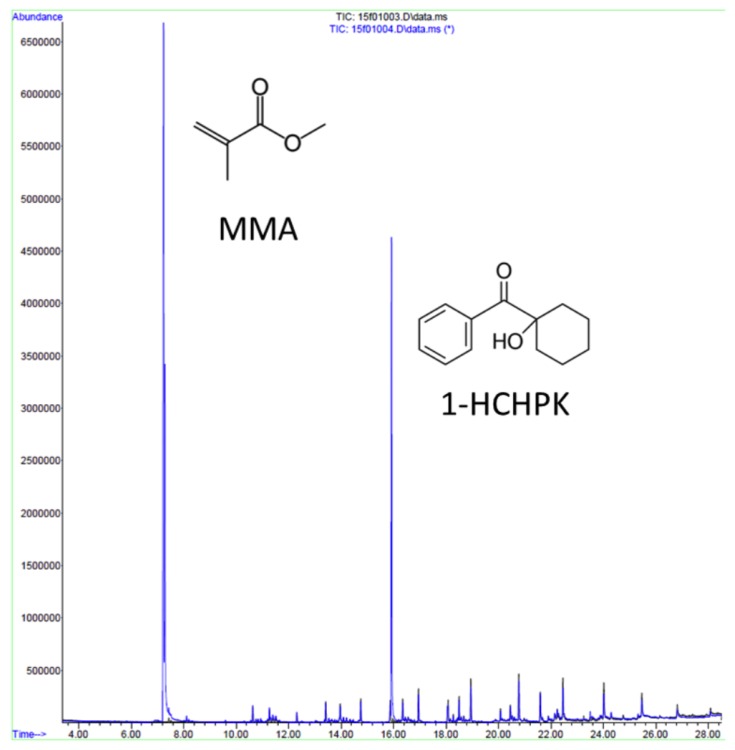
Qualitative analysis of Form Labs photopolymer leachate using gas chromatography-mass spectrometer (GC-MS) indicating the presence of photoinitiator 1-hydroxycyclohexyl phenyl ketone (1-HCHPK) and a substance closely related but not identical to methacrylate monomer.

Leaching of toxic residues to aqueous media is significantly more important for implementations of AM in bioanalytical LOC technologies. Recent reports have shown that leaching of compounds from plastic parts is relatively high in the first 24 h [30]. The rate of compound leaching is influenced partly by geometry, characteristics of resin components, and polymerization extent [36,99]. Several studies have isolated compounds such as residual monomers, additives, and photoinitiators by means of extraction with aqueous media including distilled water, natural or artificial saliva, Ringer’s solution, and organic diluents such as methanol, ethanol, and acetone. We have recently tested a random sample of photopolymer leachate from several SLA systems using gas chromatography–mass spectrometry (GC-MS) analysis. Our qualitative data based on different retention times in GC confirmed the presence of a photoinitiator 1-hydroxycyclohexyl phenyl ketone (1-HCHPK) and a substance closely related but not identical to methacrylate monomer (Figure 3).

Interestingly, 1-HCHPK has recently been reported as toxicant leaching from polyethylene ampoules used for intravenous injections [100]. Such pilot GC-MS analysis highlighted its limited detection capability for substances that are polar and non-volatile. Indeed, subsequent toxicity profiling of identified pure compounds suggested that they were responsible for only half of the cumulative toxicity effect. This warrants additional analysis to enable quantitative and conclusive identification of compounds that leach out of the 3D-printed plastic parts. Combined, these data signify possible biological risks associated with photoinitiators use in different plastic materials.

Incomplete polymerization of photo-reactive resins amid MJ, SLA, and DLP processes can lead to greater levels of uncured and highly toxic substratum in the manufactured object, which can potentially increase the leaching rate [31]. Even under optimal conditions, the conversion of monomers to polymers is usually incomplete, and even the most efficient systems achieve approximately 55–60% of complete polymerization [101]. Early termination of polymerization may further result from a higher-than-optimal PI concentration in the resin, which over-produces reactive species, or the presence of oxygen [102]. The above issues can be further intensified by part geometry. The latter affects polymerization since initiation is due to ‘line of sight’, making shadowed areas more difficult to cure, particularly at the ‘post-curing’ stage. Since the PI, compounds are not typically indicated by the resin manufacturer, mismatch of the absorption characteristics of the resin to the emission characteristics of the chosen curing method, is a potential problem that may result in incomplete polymerization. For example, most UV photoinitiators, in particular those for cationic polymerization, exhibit very weak or no absorption at 365 nm, 400 nm, and 465 nm, which makes the MPM lamps and LEDs inefficient light sources for curing [45].

When considering AM systems such as SLA, MJ and DLP for any biological applications it is important to consider that PIs, auxiliary compounds, monomers, and short chain polymers, as well as their metabolites, may not be exhausted during or after polymerization. Furthermore, they are not entirely bound to or within the printed object [103,104]. For example, small photoinitiators of 200–250 Da used in food packaging, such as 4-methyl benzophenone (4-MBP) and isopropylthioxanthone, were found to migrate from the packaging into the food, raising significant food safety concerns [105]. The authors of Short et al. [90] found that the PI antimony, a toxic heavy metal, leached from AM objects over a 24 h period. In addition, several studies on human tissues have shown that PI metabolites were implicated in material toxicity [106].

Key metabolites of PIs are free radicals, also known as reactive oxygen species (ROS), such as peroxides and peroxy radicals [40,44]. These are implicated in the damage of DNA and proteins by oxidative stress mechanisms [107]. The pathophysiology of ageing and various age-related diseases, including chronic inflammatory of the gastrointestinal tract, diseases associated with cartilage, and other neurology disorders, have been linked to oxidative damage [108].

Recent clinical investigations of biocompatibility of AM-fabricated polymeric dental implants have shown a correlation between leachate and irritation of the oral mucosa [30]. Compounds such as MMA, formaldehyde (a degradation of a copolymer formed from oxygen and methacrylate during polymerization), and dibutyl phthalate (a plasticizer) have been detected in saliva. The authors of Schweikl et al. [109] demonstrated that methacrylate monomers, such as HEMA, leach from dental materials and induce cell apoptosis as a response to the associated DNA damage. Epoxides are highly reactive molecules that are also implicated in DNA damage, apoptosis, and carcinogenic and mutagenic effects [94]. The leached PI camphorquinone has moderate cytotoxic effects as shown in human submandibular-duct cells [110]. It has been associated with cytotoxicity and correlated with a significant increase in intracellular ROS in human pulp fibroblasts [111].

A commonly used endpoint for the determination of adverse systemic effects is acute oral toxicity in rats (Table 1, Table 2 and Table 3). This quantifies the single-dose required to kill 50% of test animals (LD_50_). Since photopolymerised resin-based materials release compounds in relatively small amounts, acute oral toxicity in rats is less relevant to the assessment of the biocompatibility of these materials compared with tests documenting sublethal endpoints [91]. The latter determines the effective concentrations, which induce a response in 50% of the test animals (EC_50_), sometimes determined along with the concentration required to kill 50% of the test organisms (LC_50_). These values allow comparisons between relative biocompatibility of materials. For example, the authors of Geurtsen [111] investigated cytotoxic effects of 35 single compounds used in stereolithographic resins, in permanent 3T3 cells, and in three primary human oral fibroblast cultures. They showed that EC_50_ values varied significantly among compounds, and that the PI 2,6-di-t-butyl-4-methylphenol, the auxiliary compound 2-hydroxy-4-methoxy benzophenone, and the PI diphenyliodonium chloride had relatively elevated cytotoxic effects.

Several recent studies have investigated the biocompatibility of parts made by vat polymerization and MJ (Table 4). These studies utilized the fish embryo toxicity (FET) test, which is an established and sensitive, phenotype-based physiological analysis of developing zebrafish (*Danio rerio*) embryos. It is a relatively non-biased approach and is used extensively in the process of elucidating organ-specific toxicity and environmental adaptations at the organ, tissue, and systems level [112]. The embryonic developmental stage is considered one of the most sensitive to environmental perturbations and can be readily applied to assess the impacts of any potential toxic effects of chemicals or solid phases.

Despite SLA and MJ parts being post-processed according to manufacturer specifications, they appear to leach compounds. The authors of Macdonald et al. [13] investigated the biocompatibility of commercial resins—VisiJet Crystal EX200, VisiJet S300, Watershed 11122 XC, ABSplus P-430, and Fototec SLA-7150-Clear—using FET and found that these resins may leach toxic compounds (Table 4). Leachate from several caused malformations during embryonic development (teratogenicity) of zebrafish embryos. The authors of Oskui et al. [17] tested the biocompatibility of a Form Clear (Form Labs, Inc., Somerville, MA, USA) resin and found that exposure to leachate lowered survival rates of zebrafish embryos and elevated rates of malformations (yolk sac edema, heart edema, embryo length deformation, spine flexures, a lack of melanophore development, and a lack of swim bladders). In a similar study, the authors of Alifui-Segbaya et al. [16] showed that 48 h of exposure to a VisiJet (3D Systems Inc., Rock Hill, SC, USA) crystal-printed part leachate had lethal, sublethal, and teratogenic effects on zebrafish embryos.

Zhu et al. [18] expanded upon the above studies and performed a battery of cell-based and whole organism bioassays to assess the biocompatibility of several polymers including Watershed 11122 XC, Dreve Fototec 7150 Clear, VisiJet Crystal, Form Clear, and VisiJet SL Clear (Table 4). This work for the first time provided an in-depth and multispecies view of potential biological implications of fabricating devices using AM technologies. The published results demonstrated that leachate from polymerized parts were toxic to vertebrates and several invertebrate model organisms. All of the zebra fish larvae exposed to MJ and SLA leachates developed complete paralysis within 5 min of exposure, indicating the effect leachates may have on the central nervous system of zebrafish larvae.

These results identified VisiJet Crystal polymer as toxic despite its being classified as a substance with favourable biocompatibility, as evidenced by United States Pharmacopeia (USP) Class VI certification [13,18,113]. However, its USP Class VI certification is dependent on specific post-curing and cleaning steps [113]. These recent studies, though, have demonstrated that compounds, which are potentially toxic, remain and can leach. Furthermore, standard and specialised post-processing steps may not be adequate for removing toxicity, which still may occur in ranges affecting LOC bioassays.

While advances in AM of microfluidic and biomedical devices empower a rapidly growing number of applications, the above studies suggest that considerable caution must be exercised to mitigate biocompatibility concerns. The AM of biomicrofluidic LOC devices may not be suitable for in vitro bioassays applications. Ongoing research is aimed at solving these biocompatibility concerns by, for example, the development of AM systems capable of using biologically compatible substrata such as PDMS and PEG-DA [114].

## 5. Methods for Mitigating Toxicity of Polymeric Resins

AM is undoubtedly an elegant approach to the fabrication of monolithic functional LOC devices. Its ability to produced LOC with integrated fluidic interconnects and functional elements such as valves has been proven. In addition, several different approaches aimed at ensuring biocompatibility of photopolymer resins have been explored. Beyond customizing resin formulas to select for less toxic compounds, carefully managed post-cleaning and post-curing processes, or coating of surfaces with various biocompatible compounds, can increase biocompatibility.

While cross-linked polymer matrix may not leach, unreacted monomers, short-chain polymers, additives and PI residues are prone to leaching when the part is in aqueous media (Table 1, Table 2 and Table 3). For this reason, dentures and orthodontic devices, made using proprietary resins, are routinely stored in water for up to 24 h (depending on the type of resin) to allow uncured compounds to be released, thus reducing unwanted side effects [30]. For microfluidic LOC and biomedical devices made using proprietary photopolymer resins, coating with PDMS, a nontoxic and transparent elastomer, has also been used to overcome the limitation of unknown surface chemistry and toxicity [114,115].

Biocompatibility increases when polymerizing and post-curing light sources, and emission regimes, are matched to meet the absorption requirements of the resin compounds [46]. To optimize polymerization, the absorption maximum of PIs present in the photopolymer resin should overlap with the wavelength emitted by the polymerizing light source [45,116]. Commonly used light sources for polymerization are light-emitting diodes (LEDs), medium pressure mercury (MPM) lamps, and the UV-light-emitting diodes (UV LEDs). LED curing units emit at two wavelength peaks (400 nm and 465 nm), MPM lamps emit at one wavelength peak (365 nm) and UV LED can emit at four wavelength peaks (320 nm, 345 nm, 365 nm, and 390 nm). The total energy (light intensity times exposure time) irradiating the surface is also essential to polymerization [117]. Thus, it is necessary that the chosen polymerizing light source emits a sufficient intensity, and at wavelengths matching the absorption range of the PIs present in the SL resin.

Differences in the structure of printed parts will influence the effectiveness of post-curing treatments. To maximize exposure of the printed parts surface to the polymerizing light source, commercial curing ovens often have a rotating inner table and several floodlights that project from the inner walls and ceiling. However, LOCs have enclosed inner channels that are likely to be shaded from polymerizing light. The degree of which will depend on the penetration depth of the light source through the part, the thickness of the part, and part geometry. Shading will result in incomplete curing and less than optimal mechanical properties. For this reason, post-curing alone may not effectively remove toxicity.

Photopolymer resins classed as biocompatible require thorough post-cleaning [113]. For example, a standard cleaning procedure for a part printed in VisiJet clear consists of soaking the part in isopropyl alcohol (IPA), brushing lightly if needed, rinsing in IPA, and air-drying, followed by 10–30 min of post-curing. Cleaning procedures required for a USP Class VI certification involve soaking the part for 20 min in IPA, followed by scrubbing, and afterward four repeat cycles of soaking the part for 5 min in IPA, again followed by scrubbing. Each repeat soak is to be conducted in fresh IPA. Subsequently excess solvent is removed from the surface of the part using clean compressed air, and the part is air-dried for a minimum of 6 h, during which the part must be flipped periodically to facilitate equal drying. After this, the part must be cured for 1 h per side in a ProJet Curing Unit.

To reduce leaching, Macdonald et al. [13] experimented with coating SLA printed parts with wax and found that the coating was only effective at delaying the onset of toxicity (by ∼40 h). The authors of van den Driesche et al. [118] removed the toxicity of parts printed by DLP in resins, E-Shell 300 and HTM140, by coating them in parylene-C, which is an ultra-thin biocompatible polymer coating. Coating parts with biocompatible hydrogels (e.g., PEG-DA) or PDMS may also be an effective strategy to remove toxicity of easily accessible photopolymeric resins.

In another attempt to mitigate toxicity of photopolymeric resins by minimizing leaching in situ, the authors of Popov and Evseev [119] experimented with post-processing SLA-printed anatomical implants with supercritical carbon dioxide and found that, without treatment, the implant caused severe inflammation, whereas, with treatment, the implant was biocompatible [119].

Commercially available resins vary in how effective post-curing and cleaning techniques are at reducing toxicity. The authors of Macdonald et al. [13] treated parts by washing them in 99% ethanol and found that this increased biocompatibility of some printed parts, such as Fototec 7150 polymers. However, VisiJet Crystal still showed toxicity after treatment and 100% mortality of zebrafish embryos at 72 h was observed. The authors of Oskui et al. [17] reduced toxicity of the Form Clear SLA polymer by post-curing each side for 30 min. However, while survival and hatching rates of embryos increased compared to those exposed to non-treated parts, the majority of larvae still had elevated rates of malformations (e.g., yolk sac and heart edema and slower swim bladder development).

To improve the safety of photosensitive polymers, alternative compounds can replace those that exhibit higher toxicity. PIs that are less toxic and less likely to leach are being developed. For example, PIs derived from grafting or condensing low-molecular-weight PIs to linear, dendritic, or hyper-branched polymers exhibit reduced leaching compared to their corresponding low-molecular-weight analogues [120]. The authors of Nguyen et al. [106] used riboflavin (Vitamin B2) as an alternative PI, and this had the effect of significantly increasing the biocompatibility of 3D-printed objects, compared to those made with other commercial PIs such as Irgacure 2959 and Irgacure 369. Acrylates, which are cytotoxic, can be replaced by less toxic methacrylates and thiol-ene systems (Table 2). In general, cationic photopolymerizable systems are characterized by decreased toxicity and are, to a degree, a viable alternative to acrylate-radical photopolymerization systems Sangermano [40]. Compounds such as methacryloxypropyl trimethoxysilane-zirconium propoxide copolymer have demonstrated biocompatibility and have been used to build microneedles [121]. As previously discussed, materials such as PDMS, ORMOCER, and PEG-DA are considered relatively non-toxic.

## 6. Outlook

The AM industry is projected to grow by 17.8% by 2025 due to the expansion of AM applications, the adoption of industry standards, the improvements in material quality, and its technological capabilities [2]. Currently, SLA and DLP AM occupy ∼32% of the market share, and FDM and SLS, ∼36% and ∼33%, respectively [20]. Photopolymerization AM (namely, SLA, MJ, and DLP) systems represent by far the largest and most attractive market segment. SLA, DLP, and MPP are AM methods that are highly relevant to LOC design and manufacture as they offer higher resolution than alternatives and can utilize optically transparent materials.

While advances in AM of micro-milllifluidic devices is enabling a rapidly growing number of applications, the studies discussed in this work suggest that caution is required when devices are fabricated for biological applications. Further improvement in resin compositions by, for example, improving the depth of curing and selecting environmentally benign compounds will mitigate toxicity and simultaneously improve print resolution. The treatment of parts with organic solvents and dedicated surface modifications has been shown to minimize toxicity in some cases. These methods can be considered as a practical, albeit limited, stopgap solution to improve biocompatibility. However, due to limited knowledge about proprietary polymers and their interactions with biological specimens, such methods lack in-depth studies evaluating their effectiveness. Moreover, solvent extraction methods during post-processing steps are inherently variable and may not remove the potential for long-term effects resulting in chronic toxicity.

To solve biocompatibility issues, ongoing research efforts aim to develop AM systems proficient at using biologically compatible substrata such as ORMOCER PDMS and PEG-DA [114,122,123,124]. Despite some recent progress, these techniques are still at early stages of development and not readily available on the market.

This review highlights that, despite the obvious advantages of AM, the leaching and toxicity mechanisms of AM photopolymers need to be clarified so that innovative AM technologies can be used to advance the LOC field. In this context, we also have to become aware of the larger picture associated with AM technologies. We expect that, with their increasingly use, many relevant questions about waste disposal and about environmental and human health effects will soon emerge. Relatively little is known about long-term impacts of AM materials on human health as well as wellbeing of the environment. New research efforts exploring the above and related issues are necessary [125].

## Figures and Tables

**Table 1:** Summary of toxicity data available for photoinitiators used in stereolithography resins.

Compound	*w/w*	Available	Toxicological Information
Phosphine oxide compounds ^1^ (Type II)	0.1–5%	FORMlabs e.g., Dental and E-Shell series	Fertility impairing effect [47], acute and chronic toxic for aquatic organisms [48], toxic effect on mouse NIH 3T3 cells [38]. Not readily biodegradable by OECD criteria [48,49]. LD_50_ Oral rat > 5000 mg/kg (OECD) [48] LC_50_ (48 h) *Oryzias latipes*—6.53 mg/L (JIS K 0102-71) [49,50] EC_50_ (48 h) *Daphnia magna*—3.53 mg/L (OECD 202) [48,50] EC_50_ (72 h) *Pseudokirchneriella subcapitata*—1.56 mg/L (OECD 201) [49]
Hydroxy- acetophenone (Type II)			Readily biodegradable (OECD 301B) [51] LD_50_ Oral Rat—2.240 mg/kg [51] LC_50_ (96 h) *Salmo gairdneri*—25 mg/L [51] EC_50_ (48 h) *Daphnia magna*—50 mg/L [51]
Benzophenone compounds ^2^ (Type II)	<10%	UV-cured inks	Causes liver hypertrophy and kidney adenoma in rats [52] EC_50_ (24 h) *Daphnia magna*—0.28 mg/L [53] LC_50_ (96 h) *Pimephales promelas*—14.2 mg/L [53] BP-3 and BP-4: LC_50_ (48 h) *Daphnia magna*—1.09 and 47.47 mg/L LC_50_ (96 h) *Brachydanio rerio*—3.89 and 633.00 mg/L
Camphorquinone		Dental resins	EC_50_ mouse fibroblasts—235 μM [30]
1-hydroxy cyclo hexyl phenyl ketone		FORMlabs Irgacure 184	LC_50_ (96 h) *Danio rerio*—24 mg/L [54] EC_50_ (48 h) *Daphnia magna*—59.3 mg/L (OECD 202) [54] EC_50_ (72 h) *Desmodesmus subspicatus*—14.4 mg/L (OECD 201) [54]
Triarylsulfonium salt (Cationic)^3^	1–10%	3D Systems	EC_50_ (24 h) *Daphnia magna*—4.4 mg/L [55] EC_50_ (48 h) *Daphnia magna*—0.68 mg/L [55]

^1^ Including diphenyl(2,4,6-trimethylbenzoyl)phosphine oxide (TPO) and bis acyl phosphine oxide (BAPO). ^2^ Including benzophenone-3 (BP-3), benzophenone-4 (BP-4) [56]. ^3^ 50% propylene carbonate and 50% mixed triarylsulfonium salts, i.e., an antimonate mixture.

**Table 2:** Summary of toxicity data available for photopolymers used in stereolithography resins.

Compound	*w/w*	Available	Toxicological Information
Acrylate monomers, Acrylate and Urethane acrylate oligomers	5–60%	FORMlabs Autodesk Envisiontec 3D Systems	Toxic or harmful to various species of fish, algae and water microorganisms [49]. Potential mutagens and a reproductive and developmental toxicant. LD_50_ Oral rat >5000 mg/kg [49] LC_50_ (96 h) *Brachydanio rerio*—10.1 mg/L (OECD 203) [70] LC_50_ (96 h) *Cyprinus carpio*—1.2 mg/L (OECD 203) [71] LC_50_ (96 h) *Pimephales promelas*—34.7 mg/L (OECD 203) [70]
Methyl methacrylate monomers ^1^, and oligomers	5–90%	FORMlabs Envisiontec Dental resin	Assessment of repeated dose toxicity indicates potential to affect the liver and kidneys as indicated in animal studies [72]. Potential mutagen, and a reproductive and developmental toxicant, aquatic toxicant, and genotoxic in mammalian cell culture [73]. LC_50_ (96 h) *Salmo gairdneri*—3.4 mg/L (OECD 203) [73] LC_50_ (96 h) *Cyprinodon variegatus*—1.1 mg/L (OECD 203) [73] EC_50_ (48 h) *Daphnia magna*—2.6 mg/L (OECD 202) [73] EC_50_ (72 h) *Selenastrum capricornutum*—3.55 mg/L (OECD 201) [73] EC_50_ (96 h) *Mysidopsis bahia*—1.6 mg/L (OPP 72-3) [73] LC_50_ (96 h) *Lepomis macrochirus*—283 mg/L * [74] LC_50_ (96 h) *Oncorhynchus mykiss*—5.2 mg/L * [75] EC_50_ (48 h) *Daphnia magna*—8.74 mg/L * [75] EC_50_ (72 h) *Pseudokirchneriella subcapitata*—5.2 mg/L * [75] LD_50_ Oral rat—7900 mg/kg * [74]
Tripropylene Glycol diacrylate		3D Systems	LD_50_ Oral rat—6800 mg/kg (OECD 401) [76] LC_50_ (96 h) *Leuciscus idus* >4.6–10 mg/L [76] EC_50_ (48 h) *Daphnia magna*—89 mg/L [76] EC_50_ (72 h) *Scenedesmus subspicatus*—65.9 mg/L [76]
Hydroxyethyl Methacrylate		Dental resins	EC_50_ (48 h) *Daphnia magna*—380 mg/L (OECD 202) [77] EC_50_ (72 h) *Selenastrum capricornutum*—836 mg/L (OECD 201) [77]
3,4-Epoxy cyclohexylmethyl 3,4-epoxy-cyclohexane carboxylate	25–60%	3D Systems	EC_50_ (48 h) *Daphnia magna*—40 mg/L [78] LC_50_ (96 h) *Oncorhynchus mykiss*—24 mg/L [78] LC_50_ Oral rats—5000 mg/kg [78]
1,6-bis(2,3-epoxy propoxy) hexane	15–30%	3D Systems	Not easily biodegradable (according to OECD-criteria) [79] EC_50_ (48 h) *Daphnia magna*—47 mg/L [79] LC_50_ (96 h) *Leuciscus idus*—30 mg/L [79] LD_50_ Oral rats—2190 mg/Kg [79]
Bisphenol A-diglycidyl dimethacrylate (Bis-GMA)		Dental resins	EC_50_ mouse fibroblasts—9.35 μM [30]
Tetraacrylate ^2,3^	30–60%	Autodesk Evisiontec FORMlabs	LC_50_ (96 h) *Cyprinus carp*—1.2 mg/L ^2^ [80] LC_50_ (96 h) *Danio rerio*—7.9 mg/L ^3^ [80]

^1^ Degrades to methacrylic acid: LD_50_ Oral rat—1320 mg/kg, LC_50_ (96 h) *Oncorhynchus mykiss*—85 mg/L [81]. ^2^ Alkox. pentaerythritol tetraacrylate. ^3^ Di(trimethylolpropane)tetra-acrylate.

**Table 3:** Summary of toxicity data available for auxiliary compounds used in stereolithography resins.

Compound	*w/w*%	Available	Toxicological Information
Butylated hydroxytoluene		Dental resins	Toxic or harmful to various species of fish, algae, and water microorganisms [96] LD_50_ Oral rat >6000 mg/kg (OECD 401) [96] LC_50_ (48 h) *Oryzias latipes*—5.3 mg/L [96] EC_50_ (48 h) *Daphnia magna*—0.48 mg/L (OECD 202) [96] EC_50_ (24 h) Protozoa—1.7 mg/L [96]
Sebacate compounds ^1^	<5%	FORMlabs e.g., Dental Envisiontec	Toxic to aquatic life with long lasting effects [49], not readily biodegradable (OECD 301B) [49,97] LD_50_ Oral rat—3230 mg/kg (OECD 423) [49,97] LC_50_ (96 h) *Lepomis macrochirus*—0.97 mg/L (OECD 203) [49,97] LC_50_ (96 h) *Oncorhynchus mykiss*—7.9 mg/L (OECD 203) [49] LC_50_ (96 h) *Brachydanio rerio*—0.9 mg/L (OECD 203) [49] LC_50_ (48 h) *Daphnia magna*—8.58 mg/L (OECD 202) [97] EC_50_ (72 h) *Pseudokirchneriella subcapitata*—1.1 mg/L (OECD 201) [97] EC_50_ (72 h) *Desmodesmus subspicatus*—1.68 mg/L (OECD 201) [49]
Methylthiophenol compounds ^2^		Autodesk	LC_50_ (96 h) *Danio rerio*—9 mg/L [80] EC_50_ (72 h) *Pediastrum boryanum*—1.7 mg/L [80] EC_50_ (24 h) *Daphnia magna*—15 mg/L [80]
Hydroquinone		Dental resins	Evidence of mutagenicity in mammal studies, toxic to aquatic life; absorption, in sufficient concentrations, leads to cyanosis [98] LC_50_ (96 h) *Oncorhynchus mykiss*—0.04 mg/L [98] EC_50_ (48 h) *Daphnia magna* 0.13 mg/L [98] EC_50_ (72 h) *Pseudokirchneriella subcapitata*—0.34 mg/L [98] LD_50_ Oral rat—367.3 mg/kg [98]

^1^ Bis(2,2,6,6-tetramethyl-4-piperidyl) sebacate, Pentamethyl-piperidyl sebacate. ^2^ 2-methyl-1-(4-methylthiophenol)-2-Morpholinopropan-1-one.

**Table 4:** Summary of recent assessments on biocompatibility of parts printed with commercially available SLA and MJ printing polymers.

Resin	Organism	Toxicological Information
VisiJet Crystal	Algae ^1^	At 24 h ∼70% growth inhibition [18].
	Flea ^2^	At 24 h 100% mortality [18]
	Rotifer ^3^	At 24 h 100% mortality [18]
	Zebrafish ^4^	Stunted growth, missing eyes, reduced pigmentation and yolk sac, abnormal shapes and also appear darker [13]. Greater than 90% mortality observed at 48 h [16] and 100% mortality observed at at 72 h [13].
Watershed 11122XC	Algae ^1^	At 24 h >90% growth inhibition [18]
	Rotifer ^3^	At 24 h ∼ 100% mortality [18]
	Flea ^2,5^	At 24 h ∼ 100% mortality [18]
Fototec 7150 Clear	Algae ^1^	At 24 h >90% growth inhibition [18]
	Rotifer ^3^	At 24 h ∼ 100% mortality [18]
	Flea ^2,5^	At 24 h ∼ 100% mortality [18]
Form Clear	Algae ^1^	At 24 h ∼60% growth inhibition [18]
	Rotifer ^3^	At 24 h ∼100% mortality [18]
	Flea ^2,5^	At 24 h ∼ 100% mortality [18]
	Zebrafish ^4^	At 72 h higher rate of mortality, malformations (yolk sac edema, heart edema, embryo length deformation, spine flexures, lack of melanophore development, and a lack of swim bladders) [17].
VisiJet Clear	Zebrafish ^4^	At 48 h >90% mortality of embryos [16].
	Algae ^1^	At 24 h >90% growth inhibition [18]
	Rotifer ^3^	At 24 h ∼ 100% mortality [18]
	Flea ^2,5^	At 24 h ∼ 100% mortality [18]
MED610/620	Zebrafish ^4^	>50% lethality [16].

^1^ Freshwater algae (*Pseudokirchneriella subcapitata*)—OECD 201 Growth Inhibition Test. ^2^ Freshwater water flea *Daphnia sp.*—OECD 202 Acute Immobilization Test. ^3^ Freshwater rotifer *Brachionus calycifloru*—ASTM E1440-91 Acute Toxicity Test. ^4^ Zebrafish *Danio rerio* embryo—OECD 236 Fish Embryo Acute Toxicity (FET) Assay. ^5^ Freshwater water flea *Ceriodaphnia dubia*—USEPA Acute Toxicity Test.

## Abbreviations

The following abbreviations are used in this manuscript:

MDPI	Multidisciplinary Digital Publishing Institute
AM	Additive manufacturing
3D	Three-dimensional
CAD	Computer-assisted design
LOC	Lab-on-a-Chip
ABS	Acrylonitrile butadiene styrene
PLA	Polylactic acid
SLA	Stereolithography
MJ	Material jetting
DLP	Digital light processing
2PP	Multiphoton polymerization
PI	Photoinitiator
UV	Ultra-violet
TEGDMA	Triethylene glycol dimethacrylate
HEMA	2-hydroxyethyl methacrylate
*w/w*	weight-to-weight
GC-MS	Gas Chromatography-Mass Spectrometry
1-HCHPK	1-hydroxycyclohexyl phenyl ketone
LED	Light-emitting diodes
MPM	Medium pressure mercury
USP	United States Pharmacopeia
PDMS	Poly(dimethylsiloxane)
PEG-DA	Poly(ethylene glycol) diacrylate
OECD	Organisation for Economic Co-operation and Development
FET	Fish embryo toxicity
EC	Effective concentration
LC	Lethal concentration
LD	Lethal dose
TPO	Diphenyl(2,4,6-trimethylbenzoyl)phosphine oxide
BP-3	Benzophenone-3
BP-4	Benzophenone-4
BAPO	Bis Acyl Phosphine oxide
MMA	Methyl methacrylate
UDMA	Urethane dimethacrylate
ROS	Reactive oxygen species
MW	Molecular weight
IPA	Isopropyl alcohol
